# PIWI interacting RNAs perspectives: a new avenues in future cancer investigations

**DOI:** 10.1080/21655979.2021.1997078

**Published:** 2021-12-09

**Authors:** Pooneh Mokarram, Maryam Niknam, Mohammadamin Sadeghdoust, Farnaz Aligolighasemabadi, Morvarid Siri, Sanaz Dastghaib, Hassan Brim, Hassan Ashktorab

**Affiliations:** a Autophagy Research Center, Shiraz University of Medical Sciences, Shiraz, Iran; b Department of Biochemistry, Shiraz University of Medical Sciences, Shiraz, Iran; c Department of Internal Medicine, Mashhad Medical Sciences Branch, Islamic Azad University, Mashhad, Iran; d Endocrinology and Metabolism Research Center, Shiraz University of Medical Sciences, Shiraz, Iran; e Pathology and Cancer Center, Howard University College of Medicine, Washington, DC, USA; f Department of Medicine, Gastroenterology Division and Cancer Center, Howard University College of Medicine, Washington, Dc, USA

**Keywords:** Non-coding RNAs, piRNA, PIWI, cancer

## Abstract

As a currently identified small non-coding RNAs (ncRNAs) category, the PIWI-interacting RNAs (piRNAs) are crucial mediators of cell biology. The human genome comprises over 30.000 piRNA genes. Although considered a new field in cancer research, the piRNA pathway is shown by the existing evidence as an active pathway in a variety of different types of cancers with critical impacts on main aspects of cancer progression. Among the regulatory molecules that contribute to maintaining the dynamics of cancer cells, the P-element Induced WImpy testis (PIWI) proteins and piRNAs, as new players, have not been broadly studied so far. Therefore, the identification of cancer-related piRNAs and the assessment of target genes of piRNAs may lead to better cancer prevention and therapy strategies. This review articleaimed to highlight the role and function of piRNAs based on existing data. Understanding the role of piRNA in cancer may provide perspectives on their applications as particular biomarker signature in diagnosis in early stage, prognosis and therapeutic strategies.

## 1. Introduction

Despite the transcription of over 90% of the human genomes, only 1–2% of the genome is composed of genes that code proteins, while the remaining portion of the transcriptome consists of non-coding RNAs (ncRNAs) [1, D.-Y. 2]. ncRNAs play a pivotal role in the development and homeostasis [3]. In particular, frequent deregulation of ncRNAs in cancer can contribute to the initiation, progression, and metastasis of tumors [4, 5, Z. 6]. There are two major ncRNA categories, including regulatory and housekeeping/structural ncRNAs (tRNAs, rRNAs, and snoRNAs). Regulatory ncRNAs are grouped into large (exceeding 200 nucleotides) and small (below 200 nucleotides) ncRNAs [7]. Large ncRNAs are primarily comprised of long non-coding RNAs (lncRNAs) as well as circular RNAs (circRNAs) [8, 9. 10]. Moreover, small ncRNAs are heterogeneous, including P-element-Induced WImpy testis (PIWI)-interacting RNAs (piRNAs), microRNAs (miRNAs), transfer RNAs (tRNAs), small interfering RNAs (siRNAs), ribosomal RNAs (rRNAs), small cytoplasmic RNAs (scRNAs), small nuclear RNAs (snRNAs), and small nucleolar RNAs (snoRNAs) [11–13]. In Table 1, we have described some of the main features of three major types of small ncRNAs, siRNAs, miRNAs, and piRNAs [14–18 J. C. 19, 20, L. 21]. Concerning small non-coding RNAs, it is noteworthy that they have a critical role in the cell growth, proliferation, apoptosis, and differentiation [22]. Moreover, their regulatory functions are performed through the Argonaute (AGO) protein family as a major component of RNA silencing complexes, which is highly maintained in all organisms [23].

**Table 1.** Characteristics of major noncoding RNAs (ncRNAs)

	piRNA	miRNA	siRNA
Structure	5′ terminal uridine or tenth adenosine and 2′ O-methyl group on the 3′ ribose	phosphorylated 5ʹ ends and hydroxylated 3ʹ ends	phosphorylated 5ʹ ends and hydroxylated 3ʹ ends with two overhanging nucleotides
Size	24–32 nt	20–24 nt	21–23 nt
Processing	Dicer-independent	Dicer-dependent	Dicer-dependent
Precursor	single-stranded RNA	double-stranded RNA	double-stranded RNA
Associated protein	PIWI subfamily proteins (mainly enriched in germline)	AGO subfamily proteins (ubiquitously expressed)	AGO subfamily proteins (ubiquitously expressed)
Organism	Eukaryotes	Eukaryotes	Eukaryotes
Source	Genomic loci (transposons, repeats, piRNA loci)	Genomic loci (miRNA loci, introns)	Exogenous siRNA: Viral or other exogenous RNAs Endogenous siRNA: Genomic loci (mRNAs, Transposons, repeats), miRNA-cleaved RNAs, bidirectional transcripts
number of loci in the human genome	>30,000	2,000	Endogenous siRNA: More than 200
Expression	Highly enriched in the germline	Broad expression in most cell and tissue types	Broad expression in most cell and tissue types
Major target genes	Transposons and other genes	Coding genes	Transposons and exogenous genes
Primary functional mechanism	Transcriptional repression, heterochromatin formation (DNA methylation), RNA cleavage	Translational repression of mRNA, mRNA cleavage	Exogenous siRNA: Cleavage of exogenous RNA endogenous siRNA:RNA cleavage

The third objective of Sustainable Development Goals (SDGs) (https://www.un.org/sustainabledevelopment/sustainable-development-goals/), that were adopted by United Nations member states in 2015 to be achieved by 2030, is to ensure healthy lives and promote well-being for all at all ages. Cancers are among the main noncommunicable diseases that are regarded as important targets for SDGs. Cancers have a significant health burden, and their incidence rates have increased worldwide since last decades [24]. Efforts for finding new effective cancer biomarkers have become an integral part of cancer research because they have multiple clinical implications in different stages of disease [25–27, S. 28–31].

Cancer detection at early stage and treatment of metastatic and chemoresistant tumors need specific and effective biomarkers [25,27,32,33]. Recently, the role of piRNAs as potential biomarkers and therapeutic tools in cancers had been attracting much attention. In this review, biological and clinical aspects of piRNAs in cancer have been reviewed.

### 1.1. An overview of piRNAs

In 2001, Aravin et al. identified the presence of piRNAs as repeat-associated small interfering RNAs (rasiRNAs), derived from repeated genomic components in testis and initial embryos of *D. melanogaster* [A. A. 34, A. A. 35, 36]. However, at that time, it was unknown that those small ncRNAs were actually piRNAs. They were ultimately named piRNAs in 2006 [A. 37]. Upon this discovery in Drosophila, it was later identified that there are plenty of piRNAs in gonads of different vertebrate and nonvertebrate species such as humans and mice [38]. Immunoprecipitation and northern blot analyses demonstrated the existence of piRNAs in ovaries and testes of fetal and adult human [Z. 39].

As essential mediators in the biology of cells, piRNAs are considered as the latest, largest, and most various series of ncRNAs in somatic and germ cells [40,41]. A piRNA is a small noncoding single-stranded RNA, made up of 23–35 nucleotides, with a monophosphate group at 5′ end, a strong uracil base, and 2′-O-methylation at 3′ end, which is indicative of highly conserved functions in different species [A. 36, 37, 42, 43]. The human genome contains over 30,000 piRNA genes [14] that are mainly derived from the intergenic regions [15]. In particular, contrary to other ncRNAs, piRNAs are composed of few numbers of RNA precursors, which are long and single-stranded. Moreover, their transcription from transposons, known as ‘piRNA clusters’, is performed through a Dicer-independent pathway. These clusters are mainly situated in the peri-centromeric and sub-telomeric parts of the chromosomes [44–46].

In accordance with multiple origins, piRNAs are divided into three main types: mRNA-derived, lncRNA-derived, and transposon-derived piRNA [47,48]. Given the presence of some piRNA genes in snoRNAs and tRNAs, these can be regarded as sources of piRNAs as well [49–51]. It is noteworthy that piRNAs have a silencing impact on genes and protein regulation, PIWI-dependent transposon silencing, as well as on epigenetic regulation, germ stem cell maintenance, genome rearrangement, and reproduction and fertility regulation through attaching to PIWI proteins and forming a silencing ribonucleoprotein complex [A. A. 52–55, W. 56, 57, S. 58].

### 1.2. PIWI proteins

PIWI proteins belong to the Argonaute protein family. These proteins attach to small RNAs and are expressed in a wide range of organisms [59,60]. There are four major types of them in humans: PIWI-like protein 1 (PIWIL1 or human PIWI homolog (HIWI)), PIWIL2 (HILI (Hiwi-like)), PIWIL4 (HIWI2), and PIWIL3 (HIWI3)) [61]. These proteins are expressed in different cell types, like follicle cells and germ cells in drosophila ovaries [C. 59, 62], along with stem cells [63–66], cancer cells [J. H. 67, X. 68, 69], or even adult somatic cells [70, C. 71, 72]. Nevertheless, it is worth mentioning that PIWI expression in out-of-germline tissues is specific to certain species [73]. Additionally, PIWI proteins’ expression can be observed in a broad spectrum of body organs, such as heart, pancreas, lung, liver, kidney and brain [74,75]. The PIWI proteins comprise three major functional domains that are as follows: PIWI-Argonaute-Zwille (PAZ) domain that acts as binding site of the 3′-end of guide small RNA; the middle domain (MID) that identifies 5′-uridine (1 U-bias) of piRNAs and prescribes transposon orientation in terms of strand bound to piRNA; the PIWI-domain comprises an endonuclease region (RNase-H) which contributes to mRNA cleavage [76,77]. Traditionally, coimmunoprecipitation and subsequent sequencing or RT-PCR of RNAs revealed piRNAs binding to PIWI proteins [78]. Even though these kinds of proteins were initially found to show a crucial role in self-renewal and preservation of germ-line stem-cells (GSC) [55,79], they have been shown to be vital to piRNAs biogenesis and their chief function via transcriptional and post-transcriptional suppression of Transposable Elements (TEs) or transposons in the cytoplasm and nucleus [80].

## 2. Biogenesis of piRNAs

**Figure 1.**
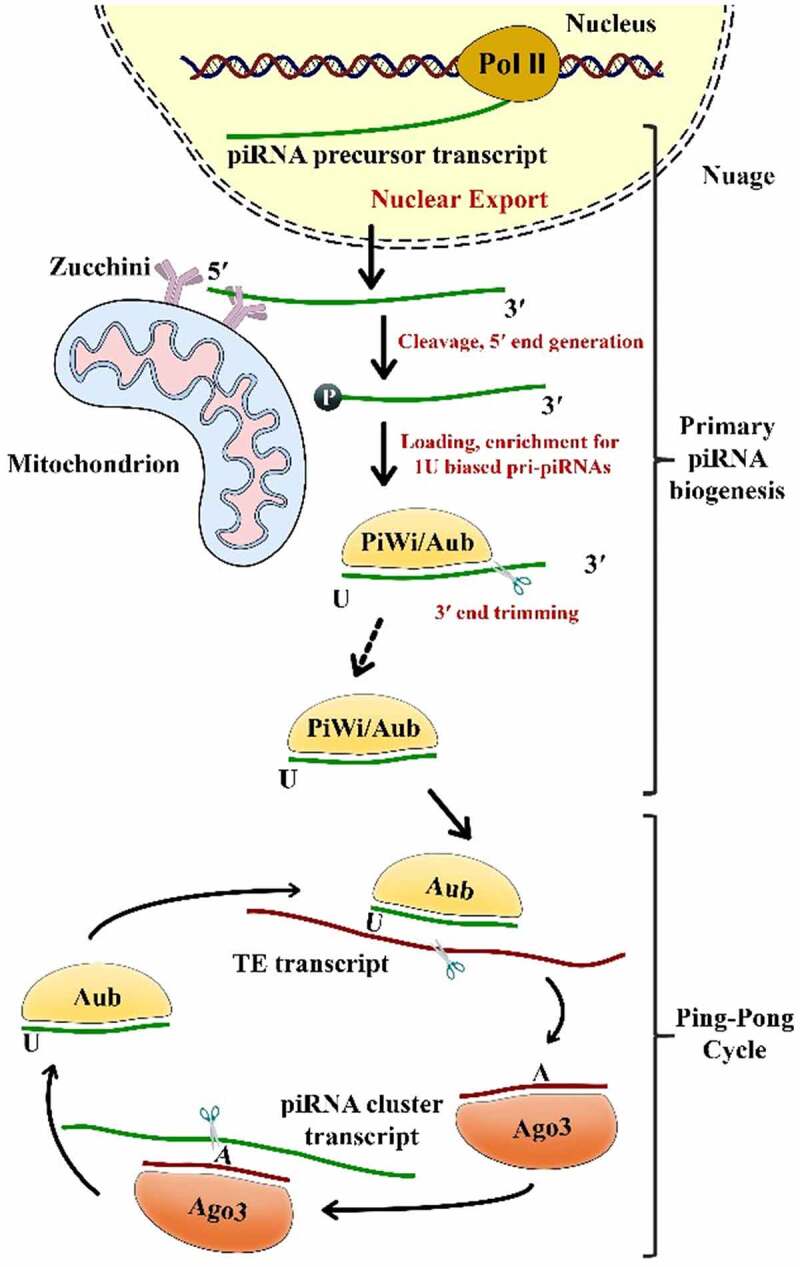
The two pathways of piRNA biogenesis: primary and secondary (Ping-Pong cycle) amplification Aub, Aubergine; Ago3, Argonaute-3; PIWI, P-element-induced wimpy testis; TE, Transposable Element

The recently emerging piRNAs need to be post-transcriptionally processed before they become entirely matured. The biogenesis of mature piRNAs comprises two main steps: the primary pathway and the ‘ping-pong’ amplification mechanism (Figure 1) [41]. The first one occurs in Drosophila’s somatic and germline cells; however, the ‘ping-pong’ cycle was only described in germline cells [7].

### 2.1. Primary amplification

In the nucleus, Riboendonuclease Zucchini (Zuc) cleaves the primary transcripts of piRNAs, to form a 5ʹ-phosphate residue*** [81]. Subsequently, PIWI is combined with a 3ʹ fragment of the transcripts and snipped to the ultimate length by a 3ʹ to 5ʹ exonuclease. It is noteworthy that the 2ʹ-hydroxy group is methylated at the 3ʹ end through a small RNA 2ʹ-O-methyltransferase enzyme, known as Hen1**** [82]. It has been shown that there is a substantial bias toward U residues on the part of the PIWI-incorporated 5ʹ-end of the piRNAs. The piRNAs attach PIWI proteins sequentially to form a piRNA/PIWI complex after they are transferred into the cytoplasmic production centers and processed into their final length [83]. Later, the complex is translocated to the nucleus where it inhibits the target gene transcription via a complementary base pairing of piRNAs with DNA. As transcriptional regulators, piRNAs recruit histone methyltransferases, thereby functioning on TE sequences via establishing heterochromatin structures [B. W. 44, 84].

### 2.2. Secondary amplification

Upon the formation of a primary piRNA, piRNA accumulation needs to be augmented by the cytoplasmic ‘ping–pong’ mechanism [54]. On the contrary to the primary pathway that correlates with PIWI proteins, piRNAs connect with AUB (Aubergine) or AGO3 proteins in order to form complexes with these proteins with complementary sequences [72]. Given its slicer activity, a primary piRNA-associated AUB present in fly germ cells identifies and cleaves active transposon transcripts. These cleavages form the 5ʹ ends of new piRNAs with a sense of orientation to transposons. Next, AGO3–piRNA complexes identify and cut cluster transcripts so that they could create antisense piRNAs subsequent to load into AGO3 and become mature through trimming or Zuc [B. W. 85, 86]. The piRNAs are amplified and gathered in the cytoplasm through a ping pong cycle that is dependent on sequence-complementary [41]. Although ping-pong characteristics have been observed in *Drosophila melanogaster*, zebrafish, and sponges, they were not identified in mice, suggesting that this pathway is present in the early evolution [87].

## 3. Biological functions of PIWI-piRNAs pathway

On account of the fact that various species have distinct piRNA sequences and functioning, piRNAs biological functions have been recognized to a limited extent. Nevertheless, according to ample evidence, piRNAs play crucial roles in genes and protein regulation, epigenetic, TEs silencing, genome rearrangement, as well as in the maintenance of homeostasis, fertilization, somatic and germline stem cell self-renewal, and embryogenesis [Y. 7, 88].

### 3.1. Retrotransposons silencing

TEs comprise a number of genetic units that are capable of moving and propagating within the genome [89]. In spite of TEs’ vital role in improving genetic diversity, they lead to genetic instability via mutations, epigenetic/genetic deregulations, and chromosomes rearrangements [90]. As an innate immune-like system, piRNAs can oppose the negative impacts of TEs on the genome to conserve the genome integrity. DNA methylation and chromatin alterations of CpG islands in germline cells to preserve normal gametogenesis are the mechanisms employed by piRNAs to silence TEs [15]. Additionally, antisense piRNAs can attach to repeat sequences of TEs and lead to their silencing through degradation of mRNAs, which comprise these repeats, and also via translational inhibition [91].

### 3.2. Genes and proteins regulation

Since piRNAs have a broad array of applications, this group of ncRNAs is recognized for its master gene expression regulatory function both in germinal and somatic cells. piRNA-mediated gene regulation is performed through a number of processes. Epigenetic mechanisms are the first one as they are involved in histone modifications and DNA methylation [92], and the other one is gene regulation mediated by piRNA at the post-transcriptional level, which is conducted through controlling mRNA stability or alternative splicing, plus interaction with RNAs or RNA endonucleolytic splitting [93]. The last point, which is of equal significance, is that interfering RNAs/piRNA-like (iRNAs/piRNA-L) are possibly involved in translational or post-translational level of gene expression via the direct connection with the protein-coding genes in physiological and pathophysiological contexts [94].

Furthermore, the first studies demonstrated that PIWI proteins participate in the regulation of translation. It has been found that at different steps of mouse spermatogenesis, mouse PIWI homolog (MIWI) and Miwi-like (MILI) are associated with the mRNA cap-binding complex, which is essential for the activation of translation [95,96]. In addition, AUB was shown to play a crucial role in translational regulation in germline stem cells of Drosophila ovaries [97]. Also, in Drosophila cultured cells, a link was found between Aub and eIF4 translation initiation factors [X. 98].

### 3.3. Differentiation

During early embryogenesis, PIWI proteins assume great significance in cell differentiation. As the main PIWI protein involved in cell differentiation by its inhibitory effect on the Transforming Growth Factor-β (TGF-β) signaling pathway, PIWIL2 has a direct interaction with Heat Shock Protein 90 (HSP90) and Mothers against decapentaplegic homolog 4 (Smad4) and impedes the formation of HSP90 – TGF-β Receptor (TβR) complex; thereby the TGF-β signaling pathway is inhibited. Moreover, PIWIL2 contributes to TβR and Smad degradation through its involvement in the upregulation of TβR ubiquitination as well as degradation by the ubiquitin E3 ligase Smurf2. Likewise, PIWI proteins assist in the differentiation of germline stem cells through post-transcriptional-level suppression of c-Fos proto-oncogene. Hence, these proteins improve piRNAs’ synthesis from 3′ UTR region of c-Fos mRNA, leading to instability of c-Fos mRNA and translation repression [99, 100, J. C. 19, 101].

### 3.4. Cell survival

Activation of numerous pro-survival molecules is enhanced by the PIWI-piRNA axis. Also, through upregulation of the anti-apoptotic molecule Fibroblast Growth Factor 8 (FGF8) expression and downregulation of pro-apoptotic p21 and Bax expression,PIWIL1 induces cell survival. Additionally, PIWIL2 mainly regulates p53 by directly interacting with c-Src and STAT3 as well as improving a PIWIL2/STAT3/c-Src complex. It would repress p53 phosphorylation and inhibit Fas-mediated apoptosis. The STAT3 activation, induced by PIWIL2, was also reported to lead to the up-regulation of the anti-apoptotic Bcl-XL [X. Zha^102^].

### 3.5. Fertilization and development

In Drosophila and murine GSCs, the PIWI-piRNA pathway is largely engaged in germline cell biology, which includes differentiation, maintenance, and function of these cells. Given their transgenerational epigenetic effects, piRNAs result in epigenetic activation of gene expression through euchromatin induction by activating H3K4me3 and inhibiting H3K27me3 in sub-telomeric heterochromatin [79,99–101,103,104].

The embryonic development, such as nuclear division, cell cycle development, chromatin organization, control of mRNA translation, chromosome integrity in pro-survival molecules during mitosis, and embryonic sex determination are the processes in which the PIWI-piRNA axis is implicated [105–107, H. 108]. During mammalian spermatogenesis and oogenesis, along with early embryogenesis, organogenesis, and post-natal, the spatial-temporal regulation and activation of piRNAs and PIWI proteins assume utmost importance [S. 58]. The regulation of PIWIL2 is performed in germline cells and contributes to genome stability maintenance by preventing gene dysregulation, chromosome rearrangements, oncogenic mutations, and TEs propagation [D.-T. 109].

### 3.6. Mitochondrial functions

piRNAs also have mitochondrial expression, wherein they originate from a sequence of 12S rRNA, tRNA, 16S rRNA, and protein-coding genes of mitochondria, including NADH-ubiquinone oxidoreductase chain 4 L (ND4L), CycloOxygenase2 (COX2), and NADH-ubiquinone oxidoreductase chain 5 (ND5) [110]. It shows that nuclear and mitochondrial piRNA transcripts have a potential cross-talk, and mitochondrial (mt)-piRNAs are involved in cell responses to oxidative stress and bioenergetics. Regarding the fact that mitochondria is an organelle involved in different cellular key processes like apoptosis and bioenergetics, functional studies are of the utmost significance to better comprehend the piRNAs role in mitochondrial epigenetics and how it affects the development of health and diseases [111].

### 3.7. Somatic functions

At the somatic level, it was reported that piRNAs are expressed in body fluids and somatic cells [112–115]. Nevertheless, their functions in the soma are still unknown because there is not enough knowledge on functional assays of piRNAs. A variety of studies have indicated the likelihood of piRNAs involvement in genes’ epigenetic regulation associated with neuronal activity, neurogenesis, plasticity [E. J. 116–118], and that pseudogenes-derived piRNAs may contribute to the regulation of parental genes [119]. Antisense piRNAs were also found to lead to regulation of immune response as well as self-tolerance genes [89]. In addition, it has been reported that sno-piRNAs exist in primary CD4 + T-lymphocytes and regulate interleukin-4 (IL-4) levels, thereby hindering their differentiation into T-helper 2 (Th2) T-cells [51].

### 3.8. Autophagic regulation

The regulatory role of piRNAs in cell cycle, apoptosis, and proliferation is becoming increasingly evident. Phosphatidylinositol 3-kinase (PI3K)/protein kinase B (AKT)/mammalian Target Of Rapamycin (mTOR) is a classical pathway that serves as a signal transduction pathway and plays a vital biological role in different cellular processes, including autophagy [120]. However, the exact role of piRNAs in autophagy needs further investigations since very limited related publications are available.

## 4. The roles of PIWI-piRNAs pathway in cancer

### 4.1. piRNAs and cancer

Given our insufficient knowledge of how piRNAs function, they have long been considered as ‘dark matter’ of ncRNAs in the genome. Nevertheless, the current document has altered our perception of piRNAs as vital clinical and biological agents in different diseases [41]. Likewise, based on recent studies, aberrant piRNAs expression might be introduced as a particular signature of cancer and could have a correlation with clinical characteristics of tumor tissues, suggesting a significant role of these molecules in a wide variety of cancers, such as colorectal, prostate, gastric, bladder, breast, lung, and hepatic cancers, as well as malignant melanoma (Table 2) [14,121,122]. Dysregulated expression of piRNAs suggests that they could be considered as tumor suppressors or oncogenes in tumorigenesis (depending on the function of their targeted gene/mRNA/protein), and as such can act as crucial regulators of the major characteristics of cancer, including cancer cell differentiation, proliferation, progression, and metastasis [93,123].

**Table 2.** Cancer-associated piRNAs

Malignancy	PiRNA	Expression	Mechanisms in cancer	References
Breast cancer	piR-932 piR-651 piR-021285 piR-4987 piR-36712 piRNA-823	Overexpression Overexpression Overexpression Overexpression Underexpression Overexpression	Stemness, EMT, and invasion Proliferation, invasion, and metastasis Increase ARHGAP11A mRNA expression and invasiveness Associated with lymph node metastasis Restrains breast cancer progression and chemoresistance by interaction with SEPW1 pseudogene SEPW1P RNA Involved in cancer stem cell regulation through altering DNA methylation	[124,142] [H. 125] [126] [L. 127] [128]
Gastric cancer	piR-651 piR-823 piR-59056	Overexpression Underexpression Overexpression	Proliferation, invasion, and metastasis Inhibit cancer cell growth, tumor-node and distant metastasis Associated with recurrence	[129] [Y.-N. 130, 205] [L. 147]
Lung cancer	piR-L-163 piR-651 piR-55490	Underexpression Overexpression Underexpression	Proliferation and invasion Proliferation, invasion, and metastasis Suppress the activation of Akt/mTOR pathway	[94,122,124]
Colon cancer	piR-651 piR-59056	Overexpression Overexpression	Proliferation, invasion, and metastasis Associated with recurrence-free survival	[Y. 131] [L. 147]
Bladder cancer	piR-ABC piR-60152	Underexpression Underexpression	Proliferation suppression, encourage apoptosis Regulate the expression of TNFSF4	[132,132]
Liver cancer	piR-Hep1 piR-823	Overexpression Overexpression	Migration and invasion Increase the production of a-SMA and COL1a1	[133,134,143]
Prostate cancer	piR-001773 piR-017184 piR-19166	Overexpression Underexpression	Promote cancer progression Inhibits migration and metastasis	[L. 135] [136]
Multiple myeloma	piR-823 piR-004800	Overexpression Overexpression	DNA methylation and angiogenesis Keeps myeloma cell survival by inhibition of apoptotic and autophagic cell death	[H. 125] [H. 137]
Glioma	piR-598	Overexpression	Promote cell proliferation	[138]

**Figure 2.**
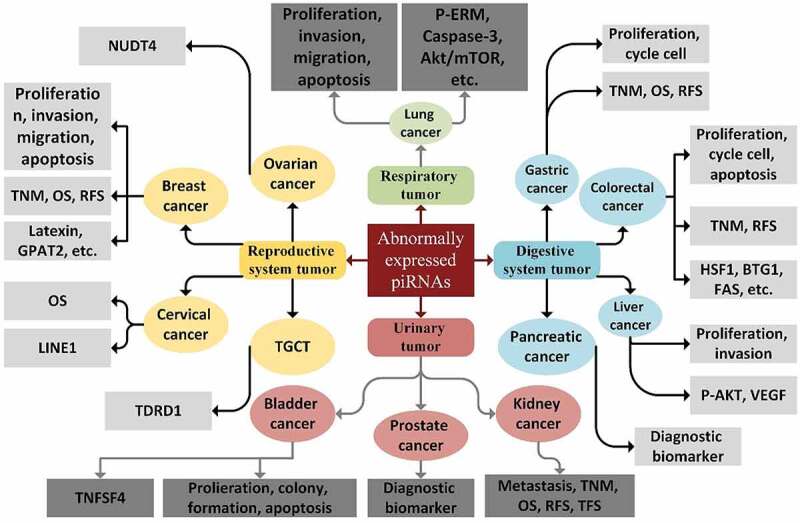
Biological functions, target genes, and clinical applications of piRNAs in cancer

The functions of piRNA pathways are shown to be associated with several processes that occur in cancer, such as genomic instability, aneuploidy, DNA methylation, cell cycle progression, repetitious expression, and cell metabolism [17, 121, Y. 139]. piRNA pathway genes have been reported to be linked to the regulation of genes that are implicated in the establishment of stem cell features [L. 140, Y. 139]. Figure 2 depicts a trace of the piRNAs in a variety of cancer types [93].

RT-qPCR analyses, next-generation sequencing and microarray screening have revealed the association between piRNAs and carcinogenesis [J. 141, 142]. As one of the most prominent discoveries, piRNAs can influence two of the most classical pathways with involvement in cancer progression, PI3K/PTEN/Akt/mTOR and Ras/Raf/MEK/ERK pathways [143]. These pathways are crucial in the regulation of a wide variety of genes, and their stimulation can enhance metabolism, growth, and survival of cancer cells [144]. Therefore, the identification of piRNAs, which are related to cancer and functional analysis of their target genes, could be capitalized on in preventive and therapeutic strategies in cancer [J. 141].

### 4.2. piRNAs as potential biomarkers and therapeutic tools

Cancers often become symptomatic when they have already spread, and this makes the treatments and interventions less effective. An effective screening biomarker must be able to distinguish the cancer at an initial stage to decrease the morbidity rate and increase the survival chances. As Table 3 shows, and in accordance with previous findings, it has been identified that aberrant piRNAs expression is associated with different characteristics of cancer patients. piRNAs are early products of different regulatory and signaling pathways. So, at least theoretically, they might be very important for early detection and treatment of cancers [41,121,145]. With a tissue-specific expression profile, piRNAs are considered as promising tissue-based diagnostic and prognostic biomarkers for cancer [146]. In addition, piRNAs are also noninvasive biomarkers that are present in exosomes or as free-circulating in serum, plasma, saliva, and stool [L. 147–151, Z. 152, X. 153]. piRNAs can be detected using microarray, sequencing, and qRT-PCR [78,146]. Alterations in circulating piRNA expression levels might be good cancer biomarkers, with higher specificity and sensitivity compared to circulating miRNAs and lncRNA-based biomarkers [L. 147]. It should be noted that no enzyme is needed for piRNA processing in the synthesis pathway. Therefore, it is beneficial to utilize synthetic piRNAs with better specificity of targets [121]. Fortunately, these small RNAs are capable of passing through the cell membrane, without degradation in the circulation and remain stable in human serum and plasma samples even after frequent freeze-thaw cycles or long-term incubation at room temperature [115,154]. This requires identifying novel piRNA biomarkers for cancer diagnosis at an early stage and their validation in independent studies with larger sample sizes and long-term clinical trials. Therefore, the combination of circulating miRNAs and piRNAs could increase its specificity and sensitivity cancer diagnosis and provide new approaches to develop a particular panel for early diagnosis and prognosis [146].

**Table 3.** Clinical applications of piRNAs in cancer

Cancer	PiRNA	Clinical application	References
Breast cancer	piR-932 piR-008114 piR-019676 piR-000552 piR-020548 piR-008113 piR-016735 piR-020450 piR-017033 piR-020365 piR-019675 piR-019914 piR-015249 piR-009294 piR-021032 piR-009051 piR-000753 piR-008112 piR-020814 piR-001318 piR-006426 piR-018,849 piR-021285 piR-017184 piR-020829 piR-019912 piR-018780	Therapeutic target Therapeutic targets	[H. 160] [161]
Gastric cancer	piR-823 piR-651	Therapeutic target and diagnostic marker Diagnostic marker	[J. 156] [L. 147]
Lung cancer	piR-651 piR-L-163 piR-55,490	Therapeutic target Diagnostic marker Therapeutic target	[D. 162, 163] [94] [L. Pe^164^]
Colorectal cancer	piR-1245 piR-015551 piR-020619 piR-020450 piR-54265 piR-823 piR-19521 piR-18849 piR-28876 piR-5937	Prognostic marker Risk assessment Diagnostic markers Valuable biomarker for screening, early diagnosis and clinical surveillance Therapeutic target Prognostic marker Prognostic marker Diagnostic marker Diagnostic marker	[129,165] [Z. 152] [166] [J. 167] [J. 168] [151]
Multiple myeloma	piR-823	Therapeutic target	[H. 125]
Prostate cancer	piR-651 piR-823	Therapeutic targets	[169]
Liver cancer	piR-Hep1	Prognostic marker	[143]
Kidney cancer	piR-823 piR-43,607 piR-32,051 piR-39,894 piR-38,756 piR-30,924 piR-51810 piR-57125 piR-34536	Prognostic marker Prognostic markers Prognostic marker Prognostic marker Prognostic marker	[149] [Y. 131] [170] [C. 10] [171] [C. 10]
Papillary thyroid carcinoma	piR-13643 piR-21238	Diagnostic markers	[172]
Diffuse Large B-Cell Lymphoma(DLBCL)	PiR-30473	Prognostic marker	[H. 173]

**Table 4.** Cancer-related PIWI Proteins

PIWI Proteins	Cancer	Expression	Function	References
PIWIL1/HIWI	Seminoma Cancer Colorectal cancer Breast cancer Gastric cancer Lung cancer Ovarian cancer Glioma Sarcomas Endometrial cancer Hepatocellular carcinoma Cervix cancer	Increased Increased Increased Increased Increased Increased Increased Increased Decreased Decreased Increased	Encourage cell propagation Inhibit migration and invasion Increase cell proliferation and stemness Encourage cell proliferation CSC self-renewa1 Migration and invasion Increase cell propagation Genomic instability Related to DNA hypermethylation Reduce cell proliferation and migration Migration and invasion	[61,124,170,182–190,190–192]
PIWIL2/HILI	Colon cancer Liver cancer Breast cancer Gastric cancer Ovarian cancer Cervix cancer Esophageal cancer Soft-tissue sarcoma	Increased Increased Increased Increased Increased Increased Increased Decreased	Proliferation, migration, and invasion Apoptosis inhibition Proliferation, apoptosis inhibition, migration, and invasion Related to poor prognosis Genomic instability Inhibit apoptosis Related to poor prognosis Reduce cell proliferation and increase apoptosis	[193, Z. 194] [J. H. 67, 195] [J. H. 196, H. 160] [175,185,190,197,198]
PIWIL3/HIWI3	Breast cancer Gastric cancer	Increased Increased	Prognosis relevance Proliferation, migration and invasion	[143,199]
PIWIL4/HIWI2	Cervical cancer Lung cancer Colon cancer Breast cancer	Increased Increased Increased Increased	Impair apoptosis, promote invasion Proliferation Migration and invasion Prognosis relevance	[D.-W. 200, X. [201] [L. 202] [143]

Despite the fact that we know little about the molecular mechanisms of piRNAs functioning in tumorigenesis, some of their characteristics would make them ideal targets for therapeutic interventions. As an example, the piRNAs’ role in DNA methylation, which results in gene silencing at the transcriptional level, is applicable in inhibiting the expression of certain oncogenes, or their functions in post-transcriptionally RNA degradation could have therapeutic advantages. Moreover, gene silencing that is mediated by RNA interference (RNAi) utilized for cancer treatment could contribute to selective silencing of oncogenic piRNAs [123]. Nevertheless, the efficiency of piRNAs as therapeutic targets is dependent on effective in-vivo delivery strategies, and prior to any application of piRNAs for therapeutic purposes, it is required that in-depth studies be conducted [155].

A number of different studies have been documented the clinical significance of piRNAs as potential biomarkers and therapeutic tools in the most common cancers. Recent studies revealed that overexpression of piR-823 in gastric cancer cells led to suppress growth of tumor, suggesting it may be considered as a promising therapeutic agent [J. 156]. In addition, serum level of piR-823 and piR-651 were significantly lower in gastric cancer patients compared to normal, highlighting its clinical value as a diagnostic biomarker [H. 157]. In lung cancer, as the most common cause of cancer death, abnormal expression of piRNAs was associated with the growth of lung cancer cells [158]. In breast cancer, the most common cancer among females, it was revealed that tumor tissues express eight piRNAs that are believed to be independent prognostic biomarkers (piR-34,736, piR-31,106, piR-36,249, piR-36,026, piR-36,318, piR-34,377, piR-35,407, and piR-36,743) [41]. Prognostic biomarkers of breast cancer are of significant importance, since local breast cancer has a 5-year survival rate of at least 90%; however, this rate declines to 60% in metastatic patients if it is regional and 30% if distant [159]. Interestingly, a cohort study documented the correlation of these breast cancer prognostic biomarkers with the survival rate. However, comparing sensitivity, specificity, and practicality of piRNAs with traditional tumor markers is a neglected area in this field.

The clinical relevance of piRNAs with cancer is not limited to the above-mentioned examples. As it is shown in Table 3, several studies reported other applications of piRNAs in the common cancers such as colorectal cancer, multiple myeloma, clear cell renal cancer, etc. Despite that, clinical researches about applications of piRNAs in targeted therapy are limited. Also, the mechanisms by which piRNA expression is altered in several cancers have not been studied yet.

### 4.3. PIWI proteins and cancer

Investigations about piRNAs have mainly been focused at the transcriptional and post-transcriptional levels, and only few researches have studied piRNA function at the post-translational level. It is noteworthy that in addition to clinicopathologic analysis, both in-vivo and in-vitro functional research has recognized all of the four human PIWI proteins as novel molecular agents involved in carcinogenesis [174]. The expression of PIWI in cancer was, for the first time, reported in seminoma [175]. Other reports on different cancers also revealed that PIWI protein expression profiles had been potentially and functionally associated with a broad range of human cancers, with both somatic and germline origin. Moreover, they have correlations with poor clinical outcomes and aggressive cancers (Table 3) [41,91,123]. Hence, these proteins were shown to be involved in the proliferation of cancer cells, metastasis, invasion, apoptosis, migration, division, and survival. As found by immunohistochemical and western blot analysis of PIWI protein expression in tissue specimens from cancer patients, PIWI proteins could be proposed as promising biomarkers for cancer prognosis and diagnosis [Y. 45, 88, 176, Y. 177].

Since the expression of PIWI proteins is not associated with the presence of large amounts of piRNAs, the mechanisms of their actions in cancers are still debated [178]. In addition, it has been reported that PIWI proteins have independent functions of piRNAs in cancer progression and metastasis. Previous studies revealed the independent function of PIWIL1 in pancreatic [F. 179] and gastric cancer [S. 180] metastasis. In mouse spermatogenesis, it has also been described that MIWI exerts an independent role of piRNAs in protein regulation [181]. Whether PIWI proteins and piRNAs affect the cancer cells independently, or they act on cancer cells together, remains a challenging issue yet to be addressed.

## 5. Database for piRNAs and functional predictions

The rapid increase in studies conducted on piRNAs has allowed for the generation of several databases, such as piRNA Bank (http://pirnabank.ibab. ac.in/) and piRBase (http://www.regulatoryrna.org/database/piRNA/) for the analysis of piRNA function, homologous piRNAs, piRNA clusters, and anticipation of the targeted RNAs [Y. 203].

Considered as the first piRNA database and a web resource about the grouped and classified piRNAs, the piRNA Bank was established by Lakshmi et al. group [204]. The database presents extensive information about 20 million identified sequences and other relevant data on reported piRNAs in human, mouse, rat, and Drosophila [41]. This above-mentioned database supports extensive search features of the organism and chromosome such as sequence homology-based search, accession numbers, name or symbol of the gene, localization on chromosomes, clusters, and corresponding repeat elements and genes. It also represents each piRNA or piRNA cluster as a graphical map of genomes (http://pirnabank.ibab.ac.in/) [Y.-N. 205]. However, it should be noted that the above-mentioned database contains only restricted amounts of piRNAs obtained from some species, and the data on the piRNA functions are rarely documented [206].

piRBase is a recently produced and special piRNA database included in RNAcentral, [K. P. 207] and is the primary database with a systematic integration of different piRNA-related data to sustain functional analysis of piRNA. In the latest release of piRBase, the unique piRNA sequences exceeded 173 million, which include 21 species*** [208]. Also, the intended mRNA records of piRNA were extended and the piRNA target lncRNAs were included*** [209]. The data concerning eight piRNAs associated cancer types such as breast, pancreas, colorectal, gastric, bladder, myeloma, kidney, and liver cancer were also appended to the recent version. It should be noted that the released piRBase v2.0 (http://www.regulatoryrna.org/database/piRNA/) introduces new web tools and enhances user interface [206].

More recently, Junyi Xin et al. constructed a user-friendly database, piRNA – expression quantitative trait locus (eQTL) (http://njmu-edu.cn:3838/piRNAeQTL/, which is also available at http://222.190.246. 206:3838/piRNA-eQTL/), using R package *Shiny*. As the first online database that provides *cis*-piRNA eQTL results via mixing genotype and piRNA expression data across 33 cancer types, it can act as a central source to find the roles of piRNAs and genetic variants in human cancer development [210].

## 6. Conclusion and future extension

piRNAs have currently been revealed to display abnormal expression in a cancer-specific manner in diverse types of cancers. PIWI proteins and piRNAs could be valid prognostic or diagnostic biomarkers in targeted therapies. As a biomarker candidate, piRNA needs to be validated by multiple centers in multiple independent, preferably prospective cohorts with large sample size, and demonstrate benefit over any existing markers. In the future, more studies and clinical trials are required to thoroughly comprehend the underlying biological mechanisms of piRNAs and their interruption. Given the continuous attempts of scientists as well as the advent of modern technologies, effective perspectives research should evaluate the association of piRNAs and cancers, which leads to an elevated piRNAs knowledge to hopefully improve cancer prevention or treatment possibilities for patients.

In spite of the importance of revealing their precise roles in cancer, there are indeed a number of unanswered questions, like whether the piRNAs’ abnormal expression explains these types of cancers, or it is a byproduct of other molecular activities. Is there a suitable threshold to differentiate healthy people from patients with specific cancer? Another question is whether metastasis, apoptosis, invasion, and proliferation of cancer cells are independently affected by piRNAs and PIWI proteins, or whether PIWI proteins and piRNAs together impact cancer cells. Likewise, as the abnormal expression of piRNA pathway could provoke stemness, analyzing the association of piRNAs and PIWI proteins with cancer stem cells is likely to establish a new course in future research about the origin of cancer. These ideas mount challenges that should be addressed before using piRNA-based treatments.

Furthermore, since piRNAs involved in cancers were clarified vastly in recent years, an unbelievable amount of data will be produced in the near future. Therefore, piRNA–cancer relationship is able to provide promising insights into piRNAs functional relationship in wide range of cancers. Novel and unique database resource will lead toward further research ideas in the field of cancer. Moreover, it is beneficial to utilize synthetic piRNAs with better specificity of targets via some piRNA target web-based prediction database resource or software based on some innovative algorithms to provide detailed information about piRNAs’ role in various cancers.

## Data Availability

Data sharing is not applicable to this article as no new data are created or analyzed in this study.

## Abbreviations

piRNAs: PIWI-interacting RNAs; PIWI: P-element Induced Wimpy testis; lncRNAs: long non-coding RNAs; ncRNAs: non-coding RNAs; circRNAs: circular RNAs; miRNAs: microRNAs; mRNAs: messenger RNAs; tRNAs: transfer RNAs; siRNAs: small interfering RNAs; rRNAs: ribosomal RNAs; scRNAs: small cytoplasmic RNAs; snRNAs: small nuclear RNAs; snoRNAs: small nucleolar RNAs; AGO: Argonaute; rasiRNAs: repeat-associated small interfering RNAs; UTRs: UnTranslated Regions; GSC: Germ-line Stem-Cells; PIWIL1: PIWI-Like protein 1; PIWIL2: PIWI-Like protein 2; PIWIL3: PIWI-Like protein 3; PIWIL4: PIWI-Like protein 4; HIWI: Human PIWI homolog; HILI: Hiwi-Like; PAZ: PIWI-Argonaute-Zwille; MID: Middle Domain; TEs: Transposable Elements; Zuc: Zucchini; AUB: Aubergine; MIWI: Mouse PIWI homolog; MILI: MIwi-Like; iRNAs/piRNA-L: interfering RNAs/piRNA-Like; eIF4: eukaryotic Initiation Factor 4; TGF-β: Transforming Growth Factor-β; HSP90: Heat Shock Protein90; Smad4: Mothers against decapentaplegic homolog; TβR: TGF-β Receptor; FGF8: Fibroblast Growth Factor 8; STAT3: Signal Transducer and Activator; ND4L: NADH-ubiquinone oxidoreductase chain 4L; COX2: CycloOXygenase 2; ND5: NADH-ubiquinone oxidoreductase chain 5; mt-piRNAs: mitochondrial piRNAs; IL-4: Interleukin-4; Th2: T helper 2; PI3K: Phosphatidyl-Inositol 3-Kinase; mTOR: mammalian Target Of Rapamycin; PTEN: Phosphatase and TENsin homolog; MEK: Mitogen-activated protein kinase; ERK: Extracellular signal-Regulated Kinase; RNAi: RNA interference; cfRNAs: circulating free RNAs; eQTL: expression Quantitative Trait Locu

## Highlights

- As early products of different regulatory and signaling pathways, piRNAs might be very important for early detection and treatment of cancers.
- Alterations in circulating piRNA expression levels might be good cancer biomarkers, with higher specificity and sensitivity compared to circulating miRNAs and lncRNA-based biomarkers.
- All of the four human PIWI proteins have been recognized as novel molecular agents involved in carcinogenesis.
